# Robust image descriptor for machine learning based data reduction in serial crystallography

**DOI:** 10.1107/S160057672400147X

**Published:** 2024-03-26

**Authors:** Vahid Rahmani, Shah Nawaz, David Pennicard, Heinz Graafsma

**Affiliations:** a Deutsches Elektronen-Synchrotron (DESY), Notkestraße 85, Hamburg, 22607, Germany; b Mid-Sweden University, Sundsvall, Sweden; SLAC National Accelerator Laboratory, Menlo Park, USA

**Keywords:** serial crystallography, data reduction, machine learning, feature extraction

## Abstract

Serial crystallography experiments at synchrotron and X-ray free-electron laser (XFEL) sources are producing crystallographic data sets of ever-increasing volume. While these experiments have large data sets and high-frame-rate detectors (around 3520 frames per second), only a small percentage of the data are useful for downstream analysis. Thus, an efficient and real-time data classification pipeline is essential to differentiate reliably between useful and non-useful images, typically known as ‘hit’ and ‘miss’, respectively, and keep only hit images on disk for further analysis such as peak finding and indexing. While feature-point extraction is a key component of modern approaches to image classification, existing approaches require computationally expensive patch preprocessing to handle perspective distortion. This paper proposes a pipeline to categorize the data, consisting of a real-time feature extraction algorithm called modified and parallelized FAST (MP-FAST), an image descriptor and a machine learning classifier. For parallelizing the primary operations of the proposed pipeline, central processing units, graphics processing units and field-programmable gate arrays are implemented and their performances compared. Finally, MP-FAST-based image classification is evaluated using a multi-layer perceptron on various data sets, including both synthetic and experimental data. This approach demonstrates superior performance compared with other feature extractors and classifiers.

## Abstract 2

This paper proposes a pipeline to categorize serial crystallography data, consisting of a real-time feature extraction algorithm, an image descriptor and a machine learning classifier. This approach demonstrates superior performance compared with other feature extractors and classifiers.

## 1. Introduction

The growth of serial crystallography at synchrotron and X-ray free-electron laser (XFEL) sources has led to an increase in the volume of crystallographic data (Chapman *et al.*, 2011 ▸). The large size of these data sets poses challenges for fast and efficient analysis, which must be overcome to enable real-time data analysis and effectively manage XFEL experiments. In serial femtosecond crystallography (SFX) experiments at the European XFEL (EuXFEL, Schenefeld, Germany), up to 3520 images per second can be recorded using an AGIPD detector (Allahgholi *et al.*, 2019 ▸), which takes images in bursts up to 4.5 MHz to match the European XFEL’s bunch structure. Despite the large-scale data sets generated by serial crystallography, only a small portion of the data may contain diffraction patterns of the target of interest. For example, a study of lysozyme using SFX at the EuXFEL generated 749 874 images in an 83 min measurement period at 150 pulses per second, of which only 25 193 images, or 3.4%, were found to contain diffraction from a protein crystal (Wiedorn *et al.*, 2018 ▸). New free-electron laser facilities, such as LCLS-II (Stanford, California, USA), will be handling experiments with even higher repetition rates, resulting in even larger data volumes (Galayda, 2018 ▸). This poses a significant challenge for the efficient processing and analysis of diffraction data in SFX experiments.

In SFX experiments, images recorded by the detector may consist of X-ray diffraction from a single-crystal ‘hit’ (or multiple crystals) or an empty shot, also known as a ‘miss’. Diffraction from a protein crystal will produce Bragg peaks. Given the experimental design of SFX, only crystal hits with Bragg peaks are valuable for further analysis. Consequently, current statistical techniques rely on peak identification to distinguish between diffraction patterns that exhibit Bragg peaks and those that only contain empty shots. Excluding empty shots leads to a significant reduction in data volume (Hadian-Jazi *et al.*, 2021 ▸, 2017 ▸; Barty *et al.*, 2014 ▸; Mariani *et al.*, 2016 ▸; Thayer *et al.*, 2017 ▸).

Traditionally, discerning bad images has been done by finding peaks using mathematical models or heuristic approaches and then rejecting images with fewer peaks than some threshold. These methods can be computationally intensive and require manual adjustment of numerous parameters, making them challenging to use for large-scale data analysis. Additionally, the performance of these methods is often limited by the quality of the data and the expertise of the user.

Recently, both traditional machine learning and deep learning techniques have shown promise in the classification and identification of patterns in images (Khan *et al.*, 2020 ▸; Naskath *et al.*, 2023 ▸). These methods can automatically learn features from large data sets, eliminating the need for manual parameter tuning. They are also able to recognize complex patterns in noisy and low-quality data, making them well suited for crystallographic applications (Rahmani *et al.*, 2023 ▸; Souza *et al.*, 2019 ▸; Ke *et al.*, 2018 ▸; Becker & Streit, 2014 ▸).

**Figure 1:**
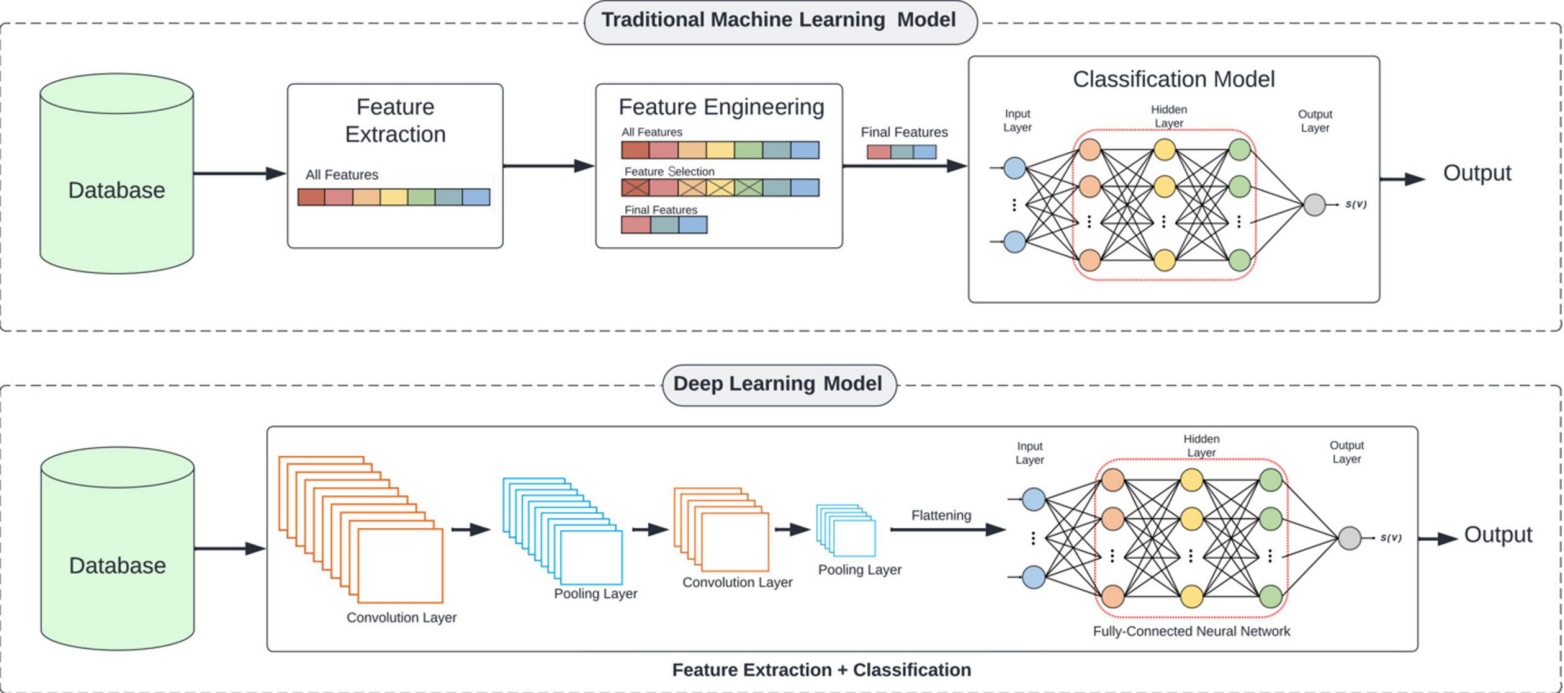
Traditional machine learning and deep learning for image classification. Both of these approaches extract key features from images and then use them to classify images by learning from training data. In traditional approaches these features are extracted using handcrafted algorithms, whereas in deep learning the features themselves are also learned during the training process.

The main difference between traditional machine learning models and deep learning models, such as convolutional neural networks (CNNs), lies in how they learn and recognize patterns in data, particularly in image recognition tasks. This is illustrated in Fig. 1 ▸. Traditional machine learning models, such as support vector machines (SVMs), rely on hand-engineered features that are extracted from the raw data and used to train the model. These features are typically designed by domain experts and involve some prior knowledge about the data. Once the features are extracted, they are fed into the machine learning model, which learns to make predictions based on the input features. In the field of image classification, multilayer perceptron (MLP) models are typically used in this way, so we group them together with traditional machine learning models in this paper. (In a multilayer perceptron, we have layers of neurons whose input weights are independent, so while a very large MLP can directly take an image as its input, this tends to be computationally unfeasible and very prone to overfitting.)

In contrast, CNNs are a type of deep learning model that can automatically learn and extract relevant features from raw data such as images. CNNs use multiple layers of filters in the form of convolutional and pooling layers. In effect, each layer in a CNN behaves like an array of neurons which all have the same weight, each ‘looking’ at a small patch of the image. This approach takes advantage of the fact that images tend to contain features that are localized, location-invariant (*i.e.* similar features appear in different parts of the image) and hierarchical. The successive layers detect low-level visual features such as edges and corners, and then combine these features into more complex visual patterns, ultimately leading to high-level recognition of objects in images. Compared with traditional feature extraction methods, CNNs tend to be more computationally intensive, both for training and inference, and they also typically require a larger amount of data to be effective. So, training and evaluating CNNs and inference may require more time and resources than with tradiational machine learning methods.

Traditional machine learning approaches can help with serial crystallography image classification by providing algorithms that can learn to recognize patterns and features in the images that are indicative of specific crystal structures. These algorithms can be trained on large sets of labelled images, and can then be applied to new images to classify them on the basis of their crystal structure. Additionally, machine learning techniques can be used to extract features from images and classify them with high accuracy, which is difficult for traditional image processing techniques. With the increase in data generated by serial crystallography, machine learning techniques can provide the necessary computational power to classify images at a high rate and with high accuracy.

Although machine learning can help with serial crystallography image classification, ‘domain gap’ and performance drop can be a serious limitation. Generally, domain gap refers to the difference in characteristics between the training and testing data sets in a machine learning model. Serial crystallography data can have substantial variability due to differences in crystal orientation, crystal size, X-ray dose and other experimental factors. Therefore, a machine learning model trained on one data set may not generalize well to new data sets with different experimental conditions, leading to a performance drop.

For example, Table 1 ▸ shows the classification performance of a CNN classifier presented by Ke *et al.* (2018 ▸) with cross-data-set training and testing for three diverse XFEL experimental data sets (denoted LN84, LN83 and LO19). The performance with data set LO19 is 93% when training and testing are completed on the same data set. However, the performance deteriorates from 93% to 91% and 74% when cross testing is performed on the LN83 and LN84 data sets, respectively. Similar results have been presented by Rahmani *et al.* (2023 ▸). This phenomenon occurs because the model has learned to recognize patterns and relationships specific to the training data, but those patterns may not apply to the testing data.

In a traditional machine learning pipeline like MLP, feature extraction is the process of identifying and extracting relevant features from the input data that will be used as inputs (feature vectors) to a machine learning model to make predictions or classify the data. Feature extraction plays an important role in mitigating the effects of domain gap and performance drop in machine learning. This is because the choice of features used by the model to represent the input data can affect the model’s sensitivity to variations in the input data, and thus its performance. For example, if the features used by the model are highly specific to the training data, such as the size and shape of particular protein crystals, the model may not generalize well to new data sets with different crystal sizes and shapes.

To address this issue, researchers have explored various feature extraction techniques, such as ORB (Rublee *et al.*, 2011 ▸), SIFT (Lowe, 1999 ▸), BRIEF (Calonder *et al.*, 2010 ▸), SURF (Bay *et al.*, 2008 ▸) and FAST (Rosten & Drummond, 2005 ▸), and also data augmentation (Shorten & Khoshgoftaar, 2019 ▸; Perez & Wang, 2017 ▸; Abdollahi *et al.*, 2020 ▸) techniques, transfer learning (Pan & Yang, 2010 ▸; Kornblith *et al.*, 2019 ▸) and feature selection (Hira & Gillies, 2015 ▸; Yang & Pedersen, 1997 ▸), to improve the robustness and generalization of machine learning models across different domains. These techniques can help to extract relevant and invariant features from the input data, and reduce the sensitivity of the model to variations in the data.

In serial crystallography images, the FAST (features from accelerated segment test) algorithm (Rosten & Drummond, 2005 ▸), which is a key-point (corner) detection algorithm, can be used to detect regions of interest (ROIs) or key-point regions. By detecting the key-point regions from an X-ray diffraction pattern, a feature vector can be generated by key-point descriptor algorithms like BRIEF (Calonder *et al.*, 2010 ▸) and used as an input vector for machine learning techniques to train a model for classifying the hit and miss images.

However, the FAST algorithm, initially designed for corner detection, requires modification to detect the centres of peaks instead of corners. This adaptation is crucial for identifying ROIs or key-point regions in the context of crystallographic analysis. The FAST algorithm is efficient, making it ideal for real-time tasks such as video processing, object tracking and robotic navigation, but it is still not fast enough for serial crystallography applications with new detectors like the AGIPD or future detectors for facilities such as LCLS-II.

In this paper, we introduce a new method called MP-FAST for detecting key points in serial crystallography images and describe a general pipeline for data classification. We implemented the MP-FAST algorithm on three different processors, a central processing unit (CPU), a graphics processing unit (GPU) and a field-programmable gate array (FPGA), and analysed their performance. CPUs are the most commonly used hardware for serial crystallography data reduction, as they are versatile and widely available. Many software packages, such as *DIALS* (Grosse-Kunstleve *et al.*, 2002 ▸) and *CrystFEL* (White *et al.*, 2012 ▸), have been developed to run on CPUs and can handle large volumes of data. However, the processing time can be lengthy, especially for large data sets, which can limit the speed at which researchers can analyse their data. GPUs offer a potential solution to the problem of processing time, as they can perform many calculations simultaneously and are highly parallelizable. Several software packages, such as GPU-accelerated *CrystFEL* and *NanoPeakCell* (Coquelle, 2022 ▸; Coquelle *et al.*, 2015 ▸), can use external modules/libraries like *pyFAI* (Kieffer *et al.*, 2023 ▸) that target GPUs, and these packages can provide significant speedups over CPU-based methods. FPGAs are a less common option for serial crystallography data reduction, but they offer potential advantages over CPUs and GPUs in terms of power efficiency and processing speed. FPGAs are highly customizable and can be optimized for specific tasks, making them well suited for specialized applications such as data reduction. However, their use requires specialized knowledge and programming skills.

The key differences between parallelization on CPUs, GPUs and FPGAs include their architecture and processing units, programming models, memory hierarchy and access patterns, and performance characteristics. CPUs are general-purpose processors with powerful cores, while GPUs are highly parallel devices with many simple cores optimized for high-bandwidth parallel processing, and FPGAs are programmable chips that can be reconfigured to perform custom logic operations. CPUs use threading for parallelization, while GPUs use single instruction on multiple data (SIMD) and FPGAs use a combination of parallel programming models. CPUs have a hierarchy of caches and RAM, GPUs have larger and faster memory optimized for parallel processing, and FPGAs have limited on-chip memory and rely on external memory. CPUs are good at handling complex tasks, GPUs excel at highly parallelizable workloads, and FPGAs are highly customizable but require more specialized knowledge to program them effectively. In the next sections, we will provide the details of our CPU, GPU and FPGA implementations.

Section 2 presents related work and Section 3 provides an overview of the original FAST key-point detection method, followed by a discussion of the modifications and enhancements made to the algorithm. Additionally, we examine various implementations of the algorithm on different platforms, including CPU, GPU and FPGA, and Section 4 covers both the experimental data and the comparison of our CPU, GPU and FPGA pipelines with other methods.

## 2. Related work

X-ray crystallography is a powerful tool for determining the structure of proteins and other biological molecules. Serial crystallography is a technique that has emerged in recent years, which involves collecting a large number of diffraction patterns from a series of small crystals. The resulting data are highly redundant and challenging to process, requiring specialized methods for indexing, integration and scaling of the data.

Data reduction in serial crystallography can be achieved in two ways: using peak finding methods based on statistical frameworks, or machine learning. Typically, peak finding methods accept or reject serial crystallography data by locating Bragg peaks and counting the total number of peaks, while machine learning methods encode key points and classify the data by learning without the need for finding peak locations. These methods can be divided into a standard machine learning pipeline with feature extractor and classifier, and data-driven methods typically known as convolutional neural networks.

Peak finding methods make use of carefully selected threshold mechanisms based on statistical frameworks to distinguish Bragg peaks from the background signal (Hadian-Jazi *et al.*, 2017 ▸, 2021 ▸; Parkhurst *et al.*, 2016 ▸). For example, Li & Zatsepin (2018 ▸) employ a global threshold mechanism to separate the background signal from the Bragg peaks. Likewise, Hadian-Jazi *et al.* (2017 ▸) identify pixels which contain potential peaks. Afterwards, the proposed approach develops a model of the local background in the neighbourhood of these potential peaks. The decision to reject the data is typically made by counting the total number of peaks. Though the process is simple, its success is often highly contingent on the selection of many input parameters. The peak finding method is a crucial component of many serial crystallography data analysis tools. For example, the *Cheetah* software suite (Barty *et al.*, 2014 ▸) has been developed to sort and filter data rapidly to achieve significant reduction. Afterwards, the reduced data are output in the facility-independent HDF5 format, enabling downstream analysis using the *CrystFEL* software tool (White *et al.*, 2012 ▸). The software suite *OnDA* (Mariani *et al.*, 2016 ▸) allows real-time monitoring of X-ray diffraction data and experimental conditions.

Existing methods for processing diffraction data require expert input parameters. At XFELS, effective communication of experimental results to various groups is crucial, along with automating data processing. The *cctbx.xfel* graphical user interface (Brewster *et al.*, 2019 ▸) is a user-friendly application that enables crystallographic scientists to efficiently navigate data reduction for serial crystallography.

Over the past decade, machine learning has produced unprecedented breakthroughs in various computer vision tasks (LeCun *et al.*, 2015 ▸). With these advancements, the crystallographic community has also made use of machine learning for various applications (Bruno *et al.*, 2018 ▸; Sullivan *et al.*, 2019 ▸; Park *et al.*, 2017 ▸; Ryan *et al.*, 2018 ▸; Wang *et al.*, 2020 ▸; Zimmermann *et al.*, 2019 ▸).

Bruno *et al.* (2018 ▸) utilized CNNs to classify the results of crystallization processes. They implemented a modified version of Inception-v3 (Szegedy *et al.*, 2016 ▸) as their model, where images were sorted into four classes: clear, precipitate, crystal and other. The data set employed in their study consisted of nearly 500 000 images, with approximately 10% of them allocated for testing purposes. Their model achieved an accuracy of around 94% on the test set.

Yann & Tang (2016 ▸) conducted an analysis of protein crystallization trial images. Their CNN approach, known as CrystalNet, demonstrated a notable improvement of approximately 8% and 20% in overall accuracy compared to the random-forest and nearest-neighbour approaches, respectively.

Ziletti *et al.* (2018 ▸) employed CNNs to classify crystal structures, specifically the arrangement of atoms within a crystal. They utilized diffraction images to represent and classify a data set consisting of approximately 100 000 crystal structures.

In a separate study, Park *et al.* (2017 ▸) focused on the classification of powder X-ray diffraction patterns using CNNs. They achieved an impressive accuracy of 94.99% in their classification tasks.

In particular, the serial crystallography community has experimented with machine learning methods to achieve data reduction (Becker & Streit, 2014 ▸; Ke *et al.*, 2018 ▸; Souza *et al.*, 2019 ▸; Rahmani *et al.*, 2023 ▸; Chen *et al.*, 2021 ▸). For example, Souza *et al.* (2019 ▸) presented a comparison of classification methods from machine learning and deep learning by curating a serial crystallography data set composed of real and synthetic images.

The concept of employing neural networks for categorizing diffraction images was introduced by Becker & Streit (2014 ▸). Their study involved investigating neural network architectures that could achieve a notable recognition rate, utilizing a minimal input layer of just three neurons. Additionally, they proposed two optimization techniques for preprocessing the data, aiming to enhance the recognition rates further, particularly for samples where a prominent disparity between signal and noise (known as CatB) exists.

Recently, neural network models have shown potential in Bragg peak finding. BraggNet (Sullivan *et al.*, 2019 ▸) was the first to demonstrate the proficiency of neural network models, particularly U-Net (Ronneberger *et al.*, 2015 ▸), in accurately segmenting peak pixels from background pixels in neutron crystallography data, including weak peaks. BraggNet used simulated peaks to create training data sets, which underwent several preprocessing steps, including centring, cropping to a specific size and adding Poisson noise.

In addition, Sullivan *et al.* (2019 ▸) observed that machine learning models performed well when training and testing are completed on synthetic data. However, the performance deteriorates considerably when such models are tested on real data. As already discussed the *Introduction* [Sec sec1], Ke *et al.* (2018 ▸) and Rahmani *et al.* (2023 ▸) made similar observations. The combination of the oriented FAST and rotated BRIEF (ORB) feature extractor and an MLP hit classifier demonstrated promising results for SFX data (Rahmani *et al.*, 2023 ▸), although those authors observed a similar performance deterioration when training and testing were performed on different data sets.

This implies that if we train a machine learning model, such as an MLP, with a particular data set and subsequently test it with a different, unseen, data set, the model is likely to experience a significant decline in performance. The observed performance drop occurs due to non-robust feature vectors. If the feature vectors exhibit robustness across diverse data sets, the model is expected to maintain consistent performance without experiencing any decline.

In the light of this issue, we here put forth a robust feature extraction algorithm for the traditional machine learning pipeline that is capable of withstanding experimental variations. Our proposed approach aims to address the limitations of existing methods and ensure consistent performance across different data sets, enabling reliable data reduction in serial crystallography experiments. By designing a pipeline that can adapt and generalize effectively, we enhance the reliability and applicability of machine learning techniques in this domain.

## 3. Pipeline

**Figure 2:**
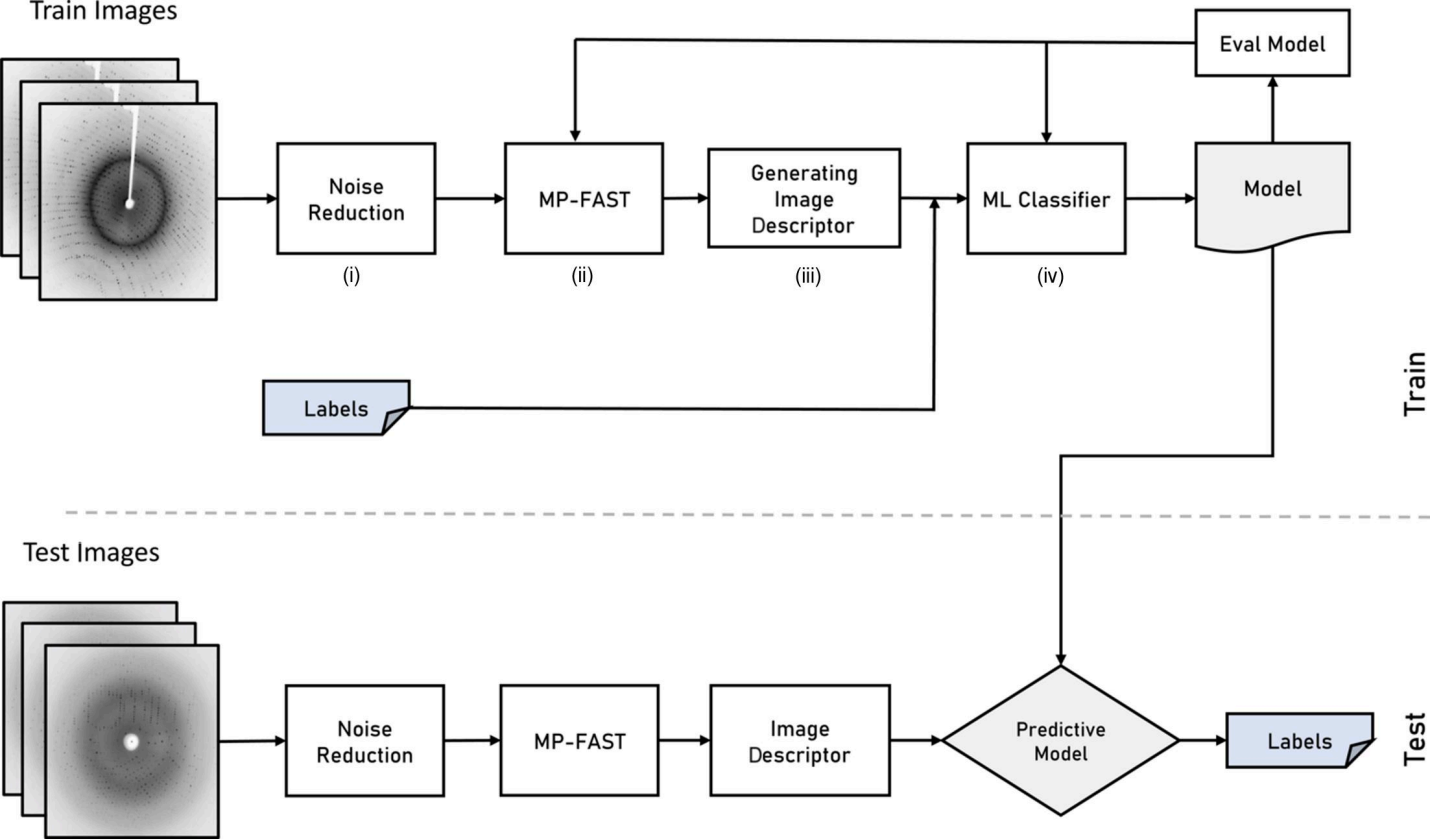
The proposed pipeline consists of four components. (i) The detector artefact and background reduction module suppress the noise and artefacts. (ii) The MP-FAST module detects key points in the image. (iii) The image descriptor vector is generated by computing the number of key features in different regions of the image. (iv) A machine learning model classifies the data into hit or miss categories.

Our objective is to use machine learning to classify X-ray crystallography diffraction data into two categories, ‘hit’ (when the X-ray beam hits a crystal) and ‘miss’ (when the X-ray beam does not hit a crystal), in order to reduce data. To achieve this, we propose a pipeline with four key components: detector artefact and background reduction, MP-FAST feature extraction, image descriptor generation and a machine learning classifier (Fig. 2 ▸). Detector artefact and background reduction are performed by populating a buffer with the most recent non-hit frames and periodically calculating a pixel-wise median through this buffer, which is used to remove the detector’s artefacts and experimental background. MP-FAST is a modified and parallelized version of the FAST (Rosten, & Drummond, 2006 ▸) key-point detection approach. The image descriptor step divides the image into *n* regions and creates a feature vector based on the number of detected key points (peaks) in each region. The generated feature vector is considered as input for the machine learning model. Finally, a machine learning classifier is trained by the computed image descriptor vectors to classify the images into data categories.

### 3.1. Background and detector artefact reduction

**Figure 3:**
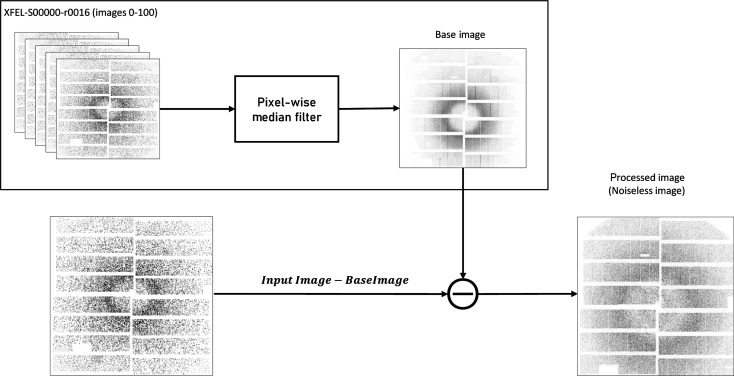
The background and detector artefact reduction pipeline.

**Figure 4:**
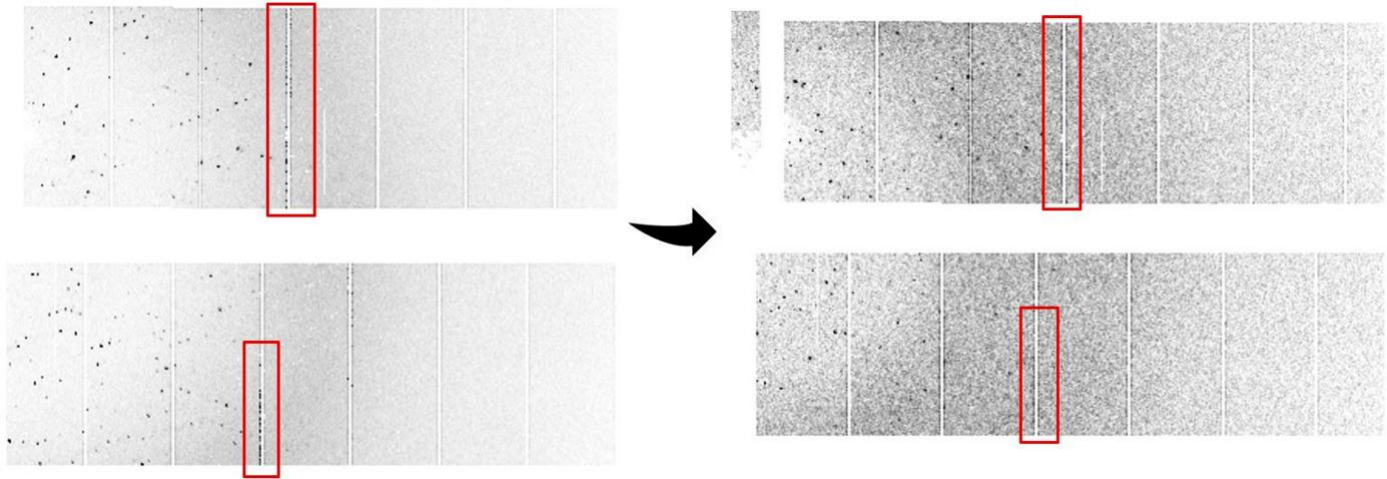
(Left) An image with artefacts. (Right) The same image after the noise reduction step.

In serial crystallography, photon detectors are used to capture diffraction images of crystals. However, these detectors can introduce noise and artefacts into the images, which can make it difficult to detect and locate the diffraction spots. These effects include fixed pattern noise, hot pixels and gain variations. Likewise, X-ray scattering from the liquid jet and beamline components will result in a background signal that can make peaks harder to discern. The background and detector artefact reduction step is designed to correct for these effects. This step is accomplished by utilizing the recent non-hit frames interspersed between hits to create an updated estimate of the background signal in the data. This is done by populating a buffer with the most recent non-hit frames and regularly calculating the pixel-wise median through this buffer, similar to the method used by Barty *et al.* (2014 ▸). This median calculation is then subtracted from new images. Fig. 3 ▸ illustrates the background and artefact reduction pipeline and Fig. 4 ▸ shows an image after this step.

A sequential median filter requires more computation time because it involves calculating the median value of each individual pixel over *n* non-hit frames. This process can be time consuming, especially for larger numbers of frames with a high resolution. Additionally, the calculation of the median requires sorting the pixel values, which is also a computationally intensive task. However, the use of modern hardware such as GPUs and parallel processing techniques can significantly reduce the computation time required for median filtering. In this paper the median filter was implemented using CUDA (an SDK released by NVIDIA for programming on their GPU architecture), *OpenCV* (https://opencv.org/) for image processing and *Thrust* (parallel sorting algorithm), which is a C++ parallel algorithms library (Hoberock & Bell, 2022 ▸).

**Figure 5:**
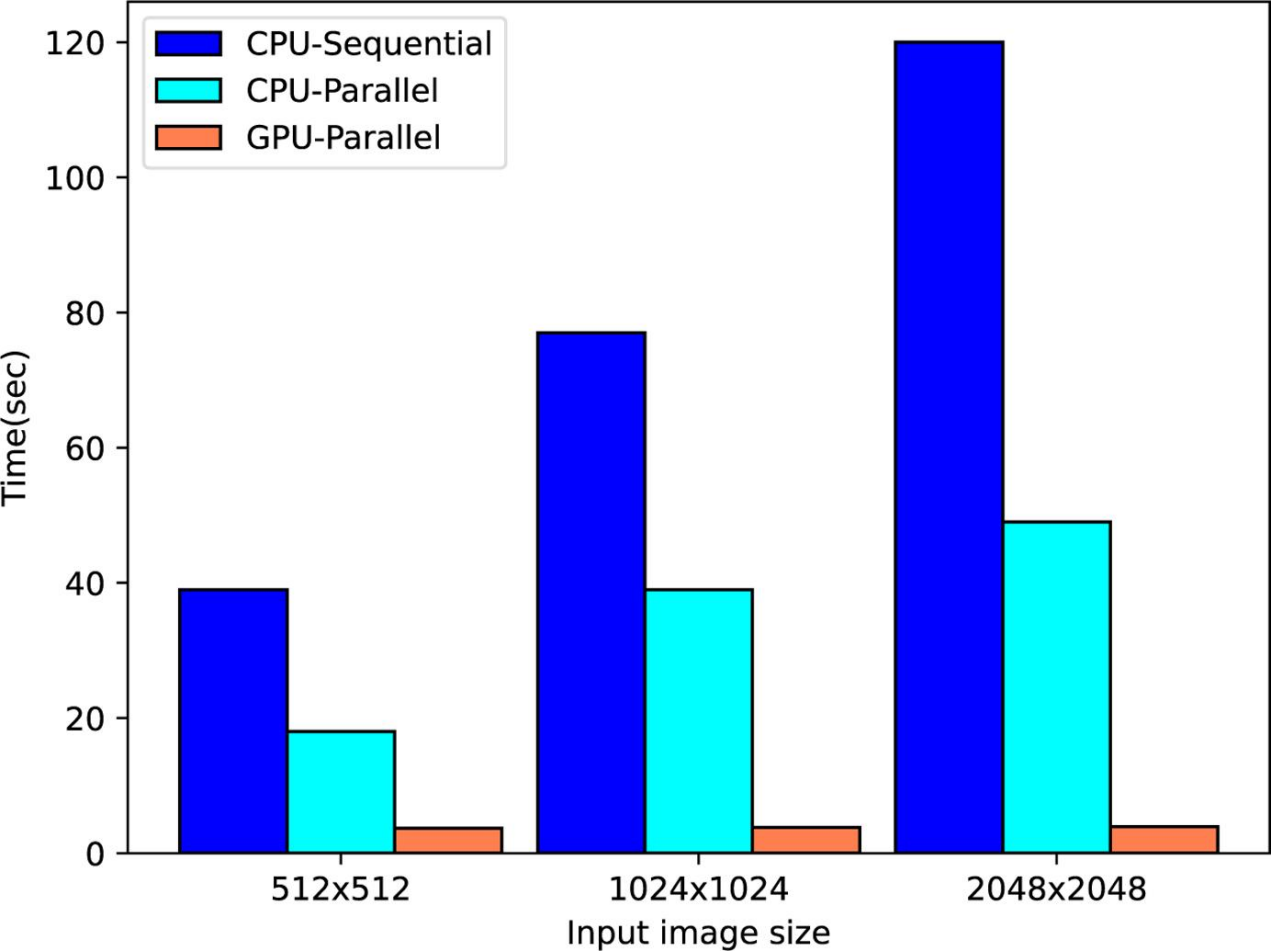
Parallel (CPU/GPU) and sequential pixel-wise median filter time performance comparison for 100 images.

This implementation was compared against a serial (*i.e.* non-CUDA) version as well as with a CUDA implementation tuned for different image sizes. Fig. 5 ▸ shows the performance times for both parallel (CPU/GPU) and sequential implementations of the pixel-wise median filter.

The CUDA threading feature is particularly relevant to parallel image filtering as it enables asynchronous programming design. Since the majority of image processing tasks involve pixel-wise filtering for a series of frames, processing them asynchronously greatly enhances program stability. Instead of waiting for serial pixel value checking, sorting and computing the median value to be completed before processing, the system can now process all pixel-wise checking in parallel. This enables multiple GPUs to be added to the system to increase the available resources.

### 3.2. Image descriptor

In traditional machine learning models like MLP, feature vectors are used to represent the input data for training the models. These vectors contain numerical values that describe the characteristics or features of the input data. Feature vectors are essential for machine learning training because they enable the models to learn from the input data and make predictions on new data. Key-point detection and feature vectors are two related concepts in computer vision and machine learning. Key-point detection refers to the process of identifying distinctive points or regions in an image that can be used for various tasks such as object detection, tracking and image stitching. These points are also known as interest points or feature points and are characterized by their uniqueness, repeatability, and invariance to scale, rotation and illumination changes. In traditional machine learning models, key-point detection is essential because it helps to reduce the dimensionality of the feature vector and removes redundant or irrelevant features that may negatively impact the performance of the model. By selecting the most important features, key-point detection improves the accuracy and efficiency of the machine learning model.

Key-point detection plays an important role in generating feature vectors for images. Typically, a set of key points is first detected in an image using algorithms such as SIFT, SURF or ORB. For each key point, a feature descriptor is computed that summarizes the local image information around the point. The collection of all feature descriptors for the key points forms a feature vector that represents the image. The choice of key-point detection algorithm and feature descriptor can have a significant impact on the quality and efficiency of feature vectors. There is a large body of research on the design and evaluation of key-point detection and feature extraction methods, and different approaches are suitable for different types of images and applications (Liu *et al.*, 2021 ▸; Rosten & Drummond, 2005 ▸).

Feature vectors can be constructed in various ways, depending on the type of data being used. For example, in the hit and miss image classification in serial crystallography, feature vectors may be constructed by extracting specific characteristics of the image, such Bragg spots. In this paper, we use the modified and parallelized FAST key-point detection algorithm, dubbed MP-FAST.

#### 3.2.1. MP-FAST

**Figure 6:**
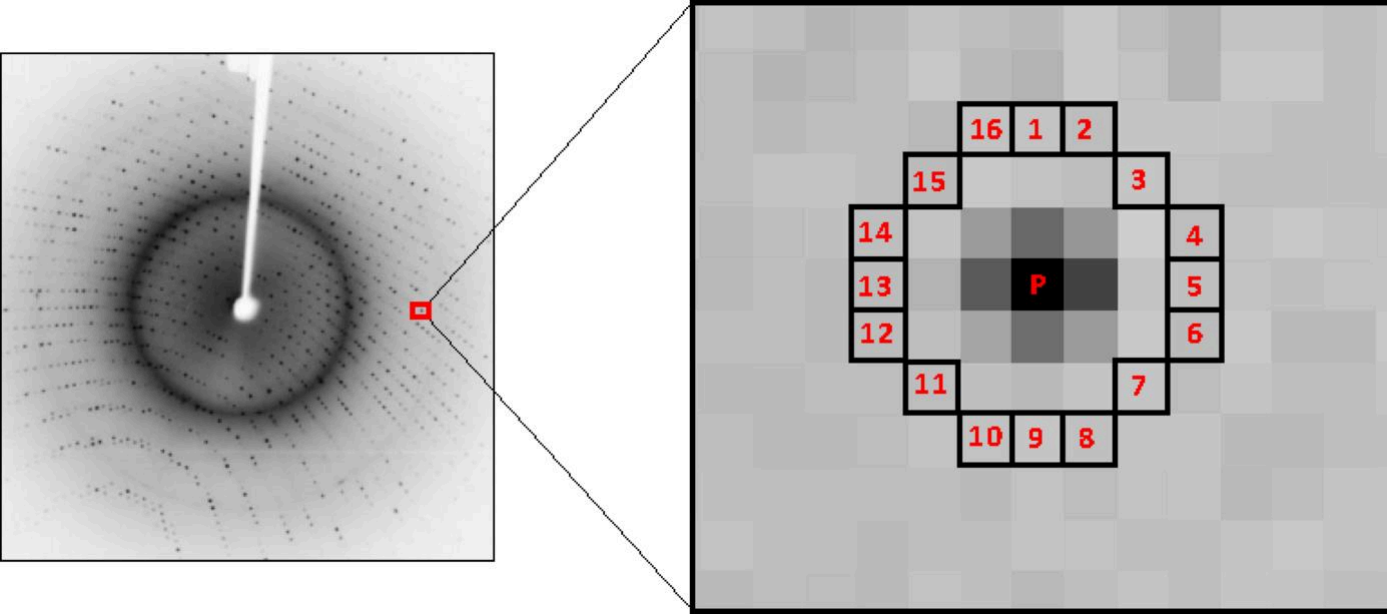
The FAST key-point detection mechanism. (*a*) A pixel *p* is compared with 16 neighbouring pixels in a circle formulation. If more than 8 pixels are darker or brighter than *p* than it is selected as a key point.

There are several feature detectors available that are highly effective, but their lack of speed makes them unsuitable for real-time applications. The FAST algorithm is useful for identifying feature points that can be utilized for object tracking and mapping in various computer vision tasks. The main concept behind the FAST algorithm is to recognize corners in an image by detecting points with a significant change in intensity in all directions. The FAST algorithm employs a circular pattern of pixels surrounding a candidate pixel to determine quickly whether it is a corner or not. This is achieved by comparing the intensity of the candidate pixel with that of the pixels in the circular pattern. If the intensity of the candidate pixel varies significantly from the surrounding pixels, it is classified as a corner. Fig. 6 ▸ shows the FAST detection mechanism.

The FAST algorithm is efficient and well suited to real-time tasks such as video processing, object tracking and robotic navigation, but it may require modification to fully meet the needs of serial crystallography experiments that use newer detectors like the AGIPD, which is capable of measuring up to 3520 pulses per second at megahertz frame rates.

**Figure 7:**
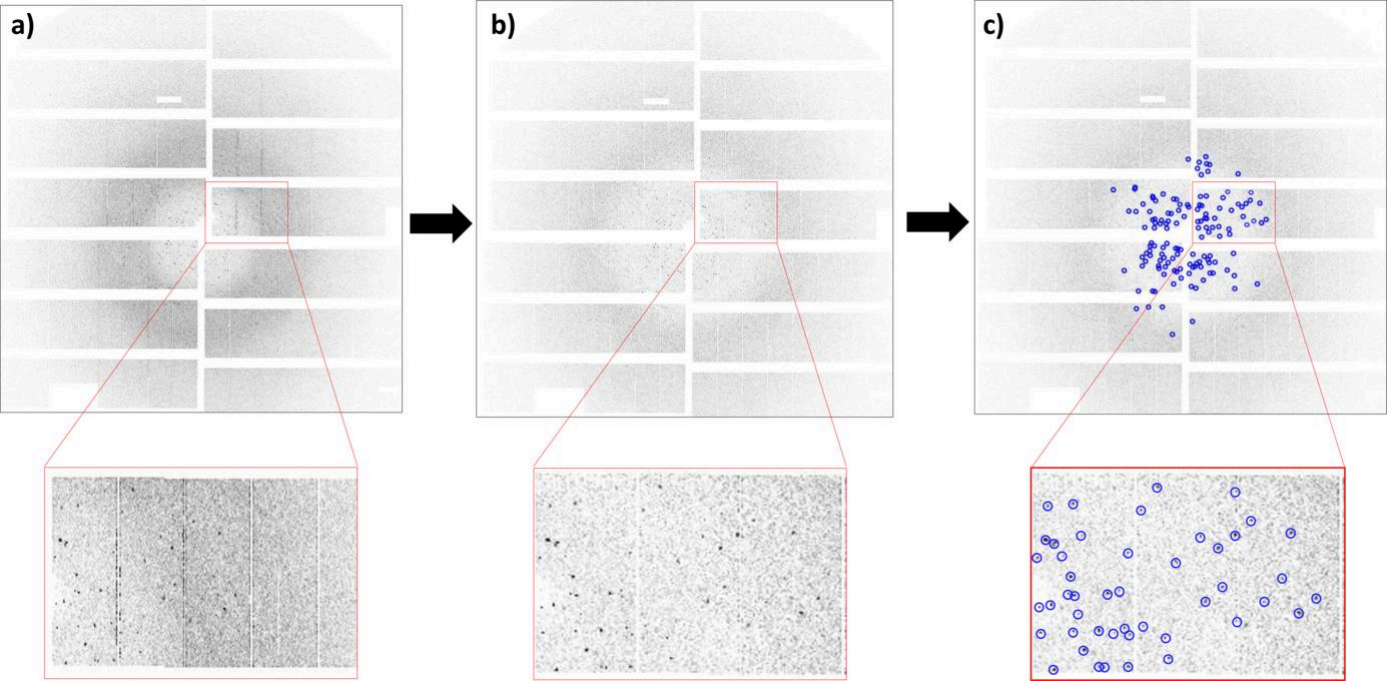
(*a*) An input image as recorded by an AGIPD detector. (*b*) The image after noise reduction. (*c*) MP-FAST key-point (peak) detection.

As mentioned above, the FAST algorithm is primarily designed for detecting sharp changes in image intensity, such as corners or edges in 2D images. On the other hand, Bragg peaks in diffraction data are characterized by a periodic pattern of intensity modulation that is determined by the crystal structure. Bragg peaks are not necessarily sharp changes in intensity, and they do not necessarily have a well defined corner-like shape. However, by applying some modifications, the FAST algorithm can be used as a detector of key points such as Bragg peaks in an image, and these key points (*i.e.* the position of the peak) can then be used to generate feature vectors for training a machine learning model (Fig. 7 ▸).

**Figure 8:**
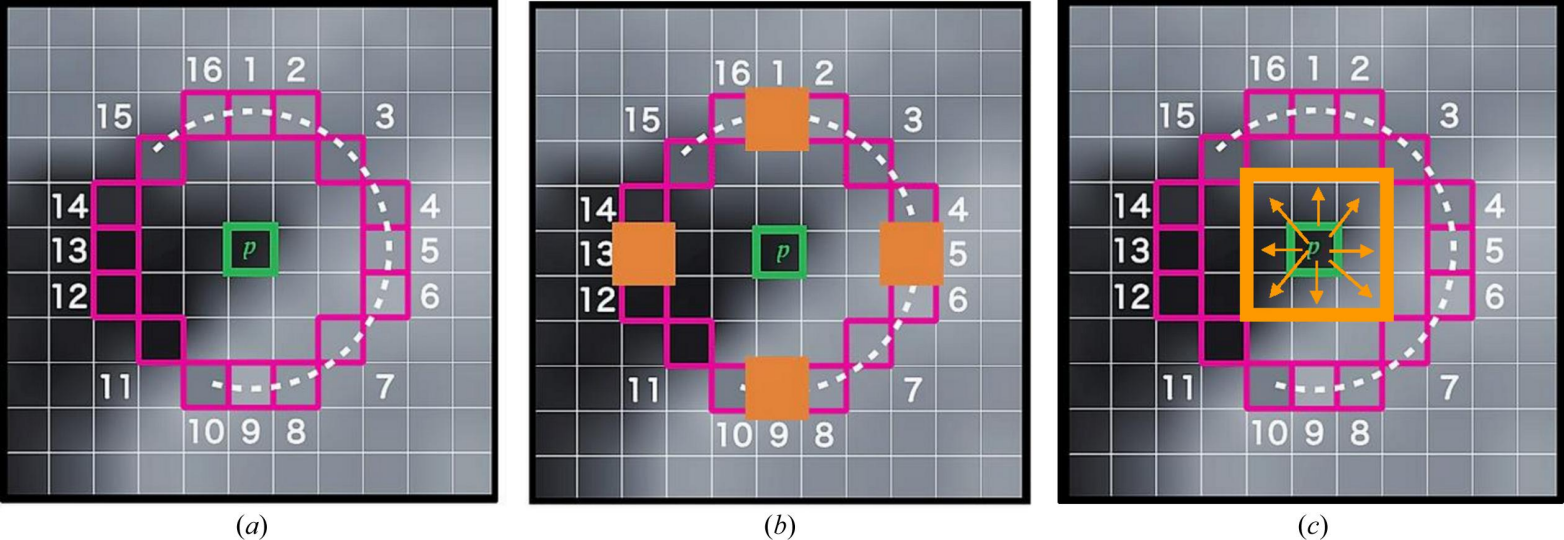
(*a*) Showing 16 pixels neighbouring a current pixel *p*. (*b*) High-speed checking for pixels 1, 5, 9 and 13. (*c*) Finding the maximum intensity for eight neighbouring pixels.

**Figure 9:**
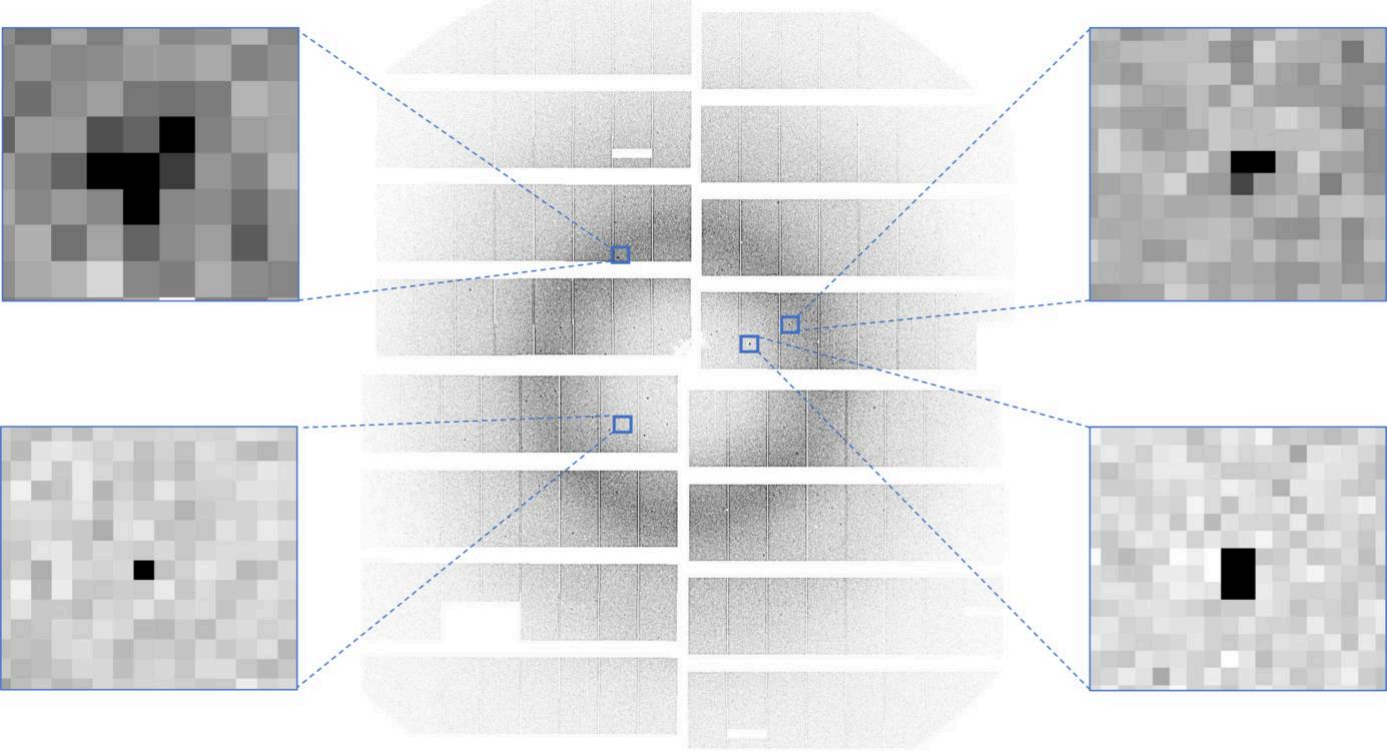
Different types of single-pixel and multiple-pixel peaks.

*Modification*. Instead of comparing pixel *p* with 16 neighbouring pixels in a circle formulation, we applied a high-speed checking mechanism. If the intensity value of the current pixel (photon counts) is less than a minimum threshold value (tr = 20) the algorithm ignores further checking of this pixel since it cannot be a peak. Otherwise, if the intensity of the current pixel is greater than the minimum threshold value tr, the intensity of the current pixel is checked versus neighbouring pixels 1, 5, 9 and 13 (Fig. 8 ▸). If the intensity of pixel *p* is greater than the intensities of at least three out of these four neighbouring pixels 1, 5, 9 and 13, then pixel *p* is considered as a candidate point for a Bragg peak. In some cases, a Bragg peak in the diffraction pattern may cover multiple pixels on the detector due to the size and shape of the crystal or the resolution of the detector (Fig. 9 ▸). This is known as a multiple-pixel Bragg peak. To detect the centre of a multiple-pixel Bragg peak accurately, we compute the maximum intensity of the eight neighbouring pixels around the candidate point. The pixel with the highest intensity is then considered as the key point for Bragg peak detection. This modified approach can improve the speed and accuracy of Bragg peak detection compared with the method presented by Rahmani *et al.* (2023 ▸). To optimize computational efficiency and prevent duplicate checking in image processing algorithms, a checking flag is typically assigned to each pixel. In this case, a checking flag value of 0 is initially assigned to all pixels in the image. When processing a candidate point, the checking flag values for the surrounding nine neighbouring pixels are set to 1. This means that the algorithm will not check those pixels again, as they have already been checked and their results have been stored. This approach can significantly reduce the computation time performance on the CPU and improve the overall performance of the algorithm. Note that for the GPU and FPGA, since all neighbouring pixels will be checked in parallel at the same time, there is no need to flag pixels.

*Parallelization on a CPU*. This can be approached from two perspectives. Firstly, by incorporating multiple cores, the CPU significantly enhances the available processing power. This means that multiple tasks can be executed simultaneously, resulting in improved overall performance.

Secondly, each core can support multiple threads of execution, which enables more efficient utilization of system resources. This is particularly beneficial when waiting for operations such as memory access. By allowing multiple threads to run concurrently on a single core, the CPU can better optimize resource allocation and maximize its computational capabilities.

In order to enhance the computational efficiency of MP-FAST on a CPU, a parallelization strategy can be employed to make full use of all available CPU cores and threads. This involves dividing the image into distinct regions, forming separate subsets of data for concurrent processing. By utilizing multiple threads, each dedicated to a specific region, computations can be executed simultaneously on different virtual cores. This parallelized approach capitalizes on the inherent parallel processing capabilities of modern CPUs, enabling a more expedited execution of the MP-FAST algorithm. Through the distribution of computational workload across various threads and cores, the system can achieve higher throughput and reduced processing times, ultimately optimizing the utilization of available hardware resources for image processing tasks.

**Figure 10:**
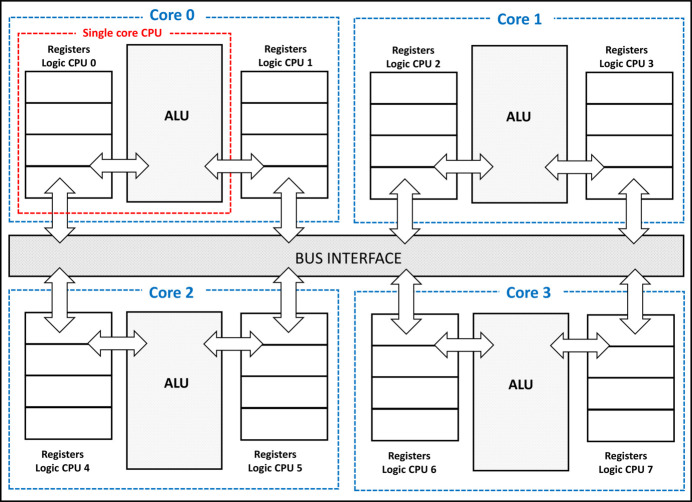
Quad-core hyperthreading in a CPU.

The number of threads used will depend on the number of physical cores and the number of hyperthreads available on the CPU. For instance, if a CPU is equipped with four physical cores, each supporting hyperthreading (*e.g.* two threads per core), it can effectively execute up to eight threads concurrently (Fig. 10 ▸). In this scenario, the image can be subdivided into eight regions, with each region assigned to a distinct virtual core. Consequently, MP-FAST can simultaneously identify key points within each region, capitalizing on the parallel processing capabilities. To fully harness the computational potential of the available threads for the MP-FAST algorithm, one strategy involves partitioning the diffraction pattern image into *N* modules, where *N* represents the number of threads, and executing the MP-FAST algorithm concurrently on each thread. This approach maximizes parallelization, optimizing the algorithm’s efficiency by distributing the workload across multiple computational units.

*Parallelization on a GPU*. GPU threads and CPU threads exhibit distinct characteristics and behaviours. In CPU architectures, individual threads commonly handle separate tasks or instruction streams autonomously. Unlike CPU threads, GPU threads are designed with parallelism in mind, allowing for simultaneous execution of tasks. They use CUDA cores (NVIDIA) or stream processors (AMD) organized into thread blocks or warps, where threads within a block/warp execute the same instructions on different data. This single-instruction multiple-thread (SIMT) model enables GPUs to process large data sets efficiently in parallel, achieving high throughput across multiple tasks.

**Figure 11:**
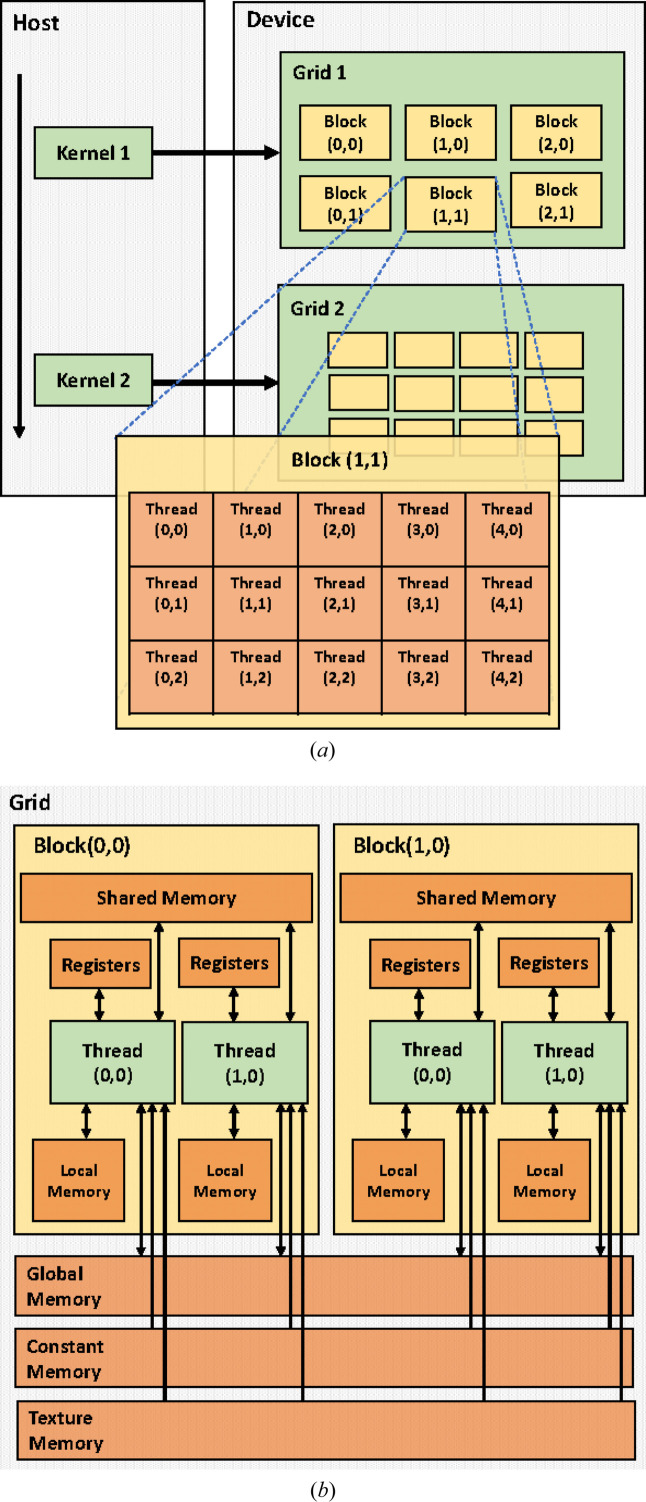
(*a*) The NVIDIA CUDA block and thread architecture. (*b*) A CUDA hardware model with global memory, constant cache, texture cache, registers and shared memory

CUDA allows developers to access the GPU’s parallel processing power to accelerate the performance of their applications. Fig. 11 ▸(*a*) shows the NVIDIA CUDA block and thread architecture and Fig. 11 ▸(*b*) shows the CUDA hardware model with global memory, constant cache, texture cache, registers and shared memory. By implementing MP-FAST on CUDA kernels, the key-point detection process can be parallelized, leading to significant acceleration compared with running the algorithm on the CPU. The algorithm is divided into several parallelizable steps. The input image is loaded into the GPU memory, and the algorithm is run on the GPU using CUDA kernels. The CUDA kernels are designed to perform the key-point detection process efficiently in parallel on multiple threads. One of the advantages of using CUDA kernels for MP-FAST is that it allows for efficient memory management. The GPU has much faster memory access than the CPU, and the CUDA kernels can be optimized to minimize the amount of data that needs to be transferred between the CPU and GPU.

In CUDA programming, a kernel is executed by launching a grid of blocks, where each block is a group of threads that run the same code in parallel. The number of blocks and threads to be used depends on the specific problem being solved and the hardware capabilities of the GPU.

To construct the CUDA kernel for MP-FAST, a block of 16 × 16 threads was utilized. Subsequently, the input image was partitioned into a grid of (*W* + 15)/16  ×  (*H* + 15)/16 blocks, where *W* and *H* are the width and height in pixels, respectively.

**Figure 12:**
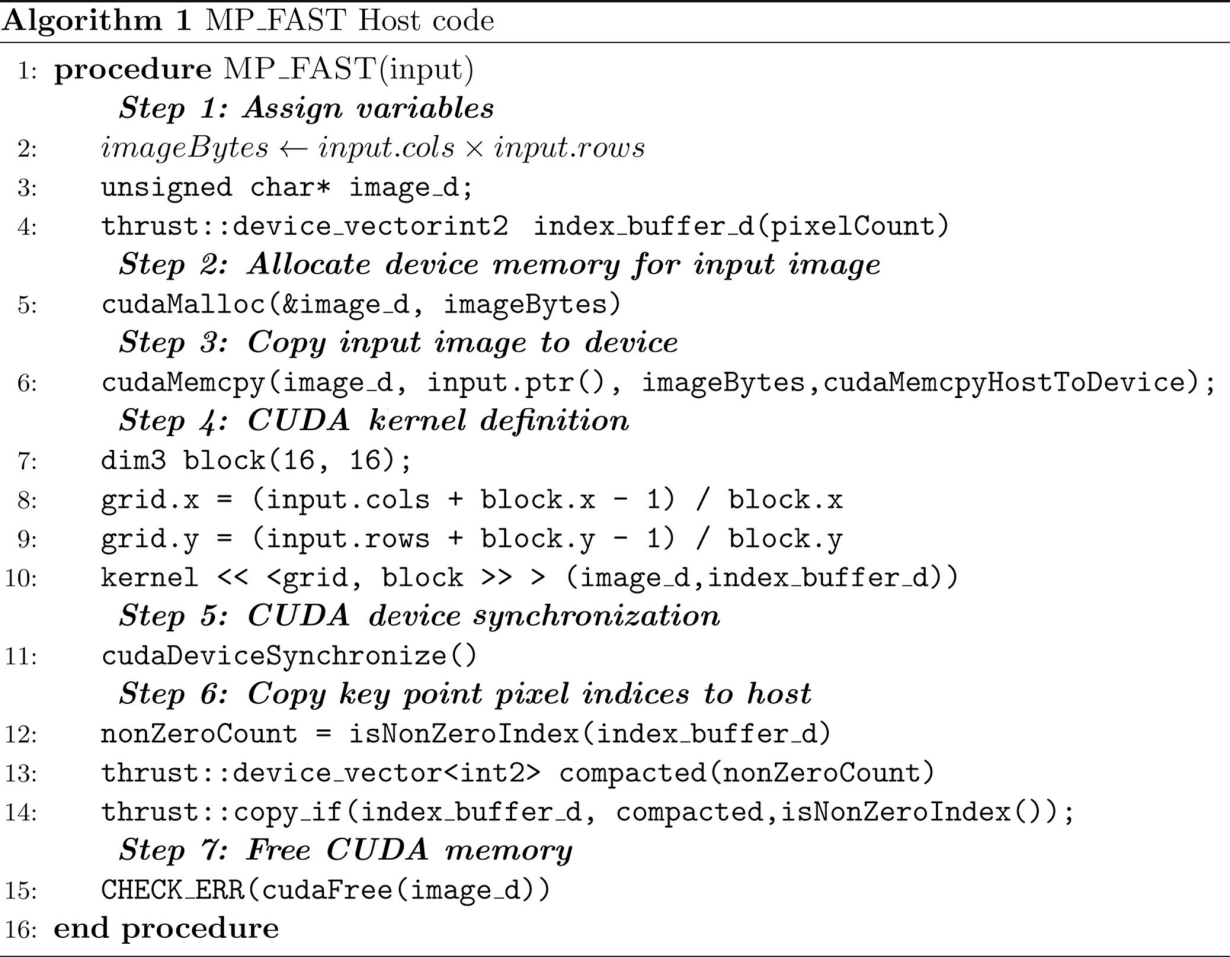
The code for Algorithm 1.

**Figure 13:**
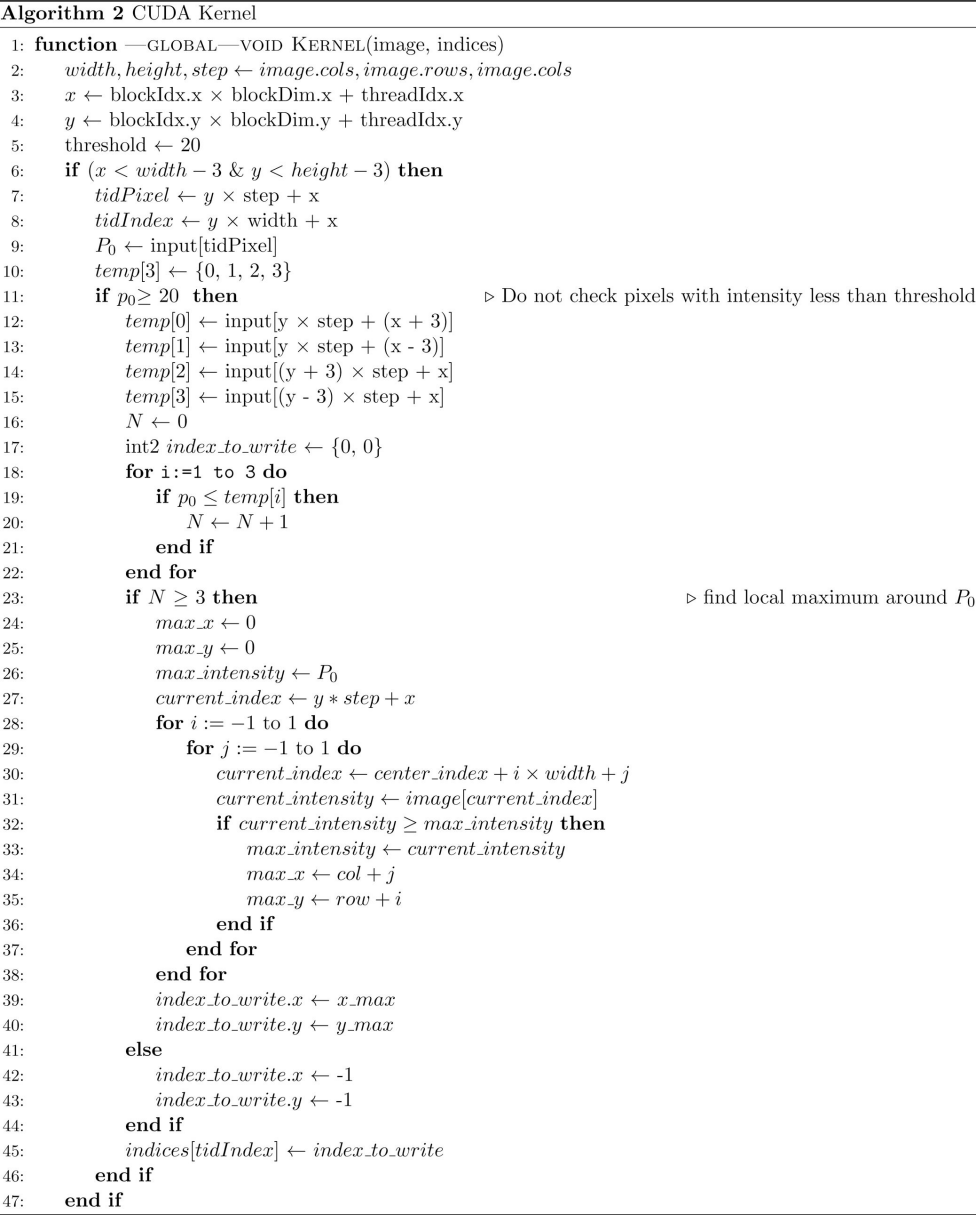
The code for Algorithm 2.

The image is transferred to CUDA global memory, and the choice of a 16 × 16 block size is primarily driven by the goal of ensuring the kernel’s successful execution. Defining a CUDA kernel involves considering certain limitations. For instance, the total number of threads per block should not exceed 512 for compute capability 1.*x* or 1024 for compute capability 3.*x* or later. The maximum block dimensions are constrained to [512 × 512 × 64] or [1024 × 1024 × 64] for compute capability 1.*x*/3.*x* or later, respectively. The shared memory usage is capped at 16 kB/48 kB/96 kB for compute capability 1.*x*/2.*x*–6.2/7.0 (NVIDIA *et al.*, 2022 ▸). Staying within these constraints ensures that any successfully compiled kernel will launch without errors. To prevent kernel crashes, we perform verify memory allocation (line 5 of Algorithm 1, Fig. 12 ▸) and index bounds checking (line 6 of Algorithm 2, Fig. 13 ▸). By integrating these practices into the CUDA code, the potential for read-access violations can be minimized, leading to enhanced robustness in GPU kernels.

*FPGA accelerator*. FPGAs are semiconductor devices that contain many blocks of circuitry of different kinds (such as combinatorial logic, digital signal processing and RAM) connected through programmable interconnects. While compiling software to run on a CPU involves translating the program into a series of instructions in machine code for the CPU to follow, to create an FPGA’s firmware we describe the functionality we need, and then a synthesis process finds a suitable configuration of blocks and interconnects that achieves this functionality. In turn, this means that FPGAs are not suited to quickly switching from one algorithm to another on the fly (since this requires reconfiguring the device), but for a given algorithm they can offer a great deal of processing power and consistent latency.

**Figure 14:**
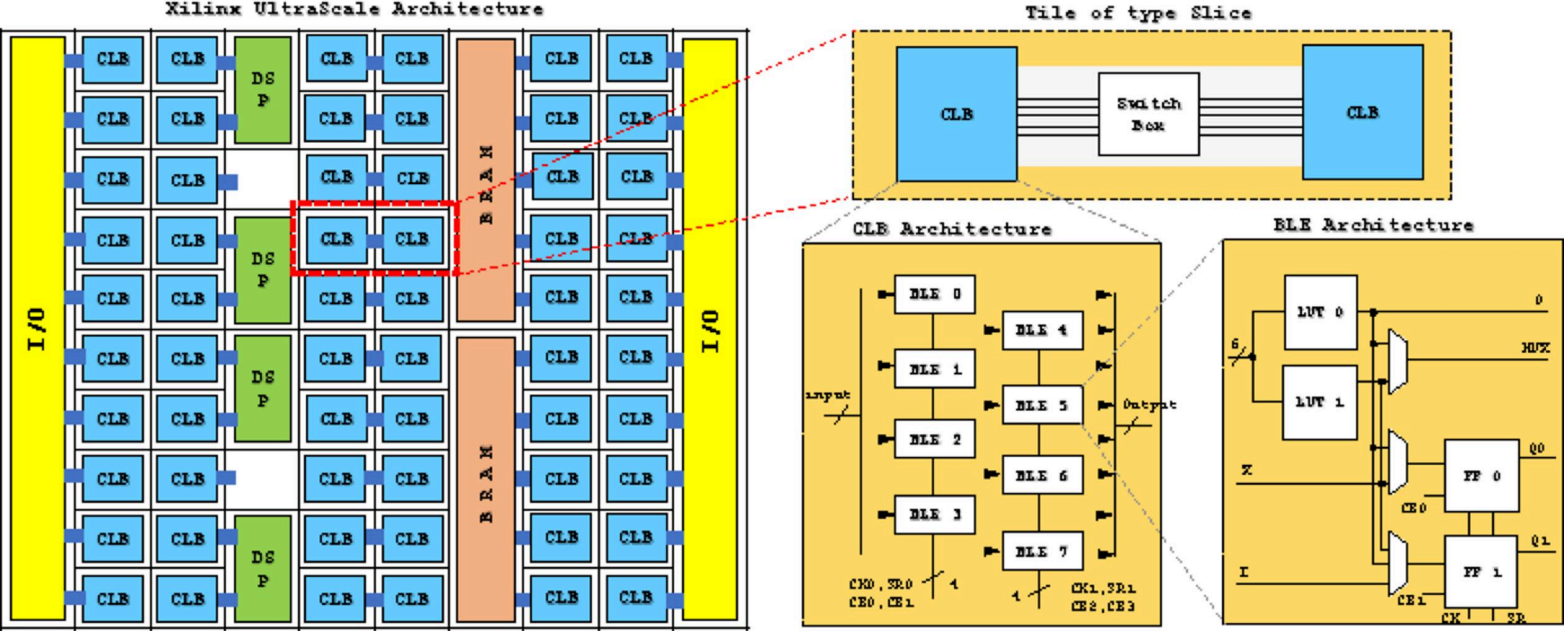
The different parts of the Xilinx UltraScale architecture. Used with permission of ACM, from Abuowaimer *et al.* (2018 ▸).

Recently, vendors like Xilinx have started offering FPGA-based data centre accelerator cards (Fig. 14 ▸). As well as the FPGA, these cards typically include built-in network links and on-board memory, and connnect to the host PC using standard interconnects like PCIe. The high-speed Ethernet ports give the potential for the card to receive data directly from the detector and process them, before passing the results into the PC’s memory. However, in this first implementation the FPGA receives data from the PC.

Unlike CPUs and GPUs, a great deal of the parallelism offered by FPGAs is due to pipelining. An algorithm can be implemented by a series of hardware blocks in the FPGA (such as digital signal processing blocks, lookup tables *etc.*) and data elements will be streamed from one block to another, having operations performed on them. This is analogous to a car being constructed on an assembly line, where at any given moment many cars are being worked on, in different stages of assembly. (Further parallelism can be achieved, for example, if each element is a vector rather than a scalar.)

For this FPGA implementation of MP-FAST, we used the Xilinx Alveo U280 card (Abuowaimer *et al.*, 2018 ▸). While FPGAs are typically programmed with hardware description languages, Xilinx offers a ‘high-level synthesis’ tool, where algorithms can be written in C++ and then compiled into firmware. Directives called pragmas can be included in the code to control how the firmware is implemented, and to help guide the compiler in finding optimizations. For example, a pragma can indicate that instructions in a loop should be implemented in a pipelined way. In turn, the structure of the algorithm can strongly affect the performance. For example, if the processing of element *K* + 1 depends on the result of processing element *K*, this may prevent pipelining, or at least create a delay of multiple clock cycles before each new element can start to be processed.

The FPGA implementation of MP-FAST was aimed at processing one pixel per clock cycle. Optimization of the algorithm required a few key elements. Firstly, the algorithm must explicitly handle how data are buffered in the FPGA during processing. When processing each pixel, we also need to access the adjacent pixels. So, a ring buffer is used to hold four lines of the image (three for processing and one for loading), with one new pixel being loaded in each clock cycle. In turn, pragmas indicate that the buffer should be implemented in a way that allows the required five pixel reads per clock cycle, and that iterations are independent (*i.e.* processing pixel *K* will not change the buffer in a way that affects pixel *K* + 1). Secondly, the logic to iterate over the pixels and control the ring buffer uses simple loops with fixed bounds where possible, to make it easier for the compiler to infer optimal pipelining. Transferring data between the host PC, the high-bandwidth memory (HBM) on the Alveo and the FPGA itself was based on examples provided by Xilinx, using the ‘load, compute, store’ pattern that they recommend (Advanced Micro Devices, https://docs.xilinx.com/r/en-US/ug1393-vitis-application-acceleration).

The time taken to finish the execution of one such kernel is 1 ms for an image size of 512 × 512 and 3.02 ms for an image size of 1024 × 1024. Resource usage in the FPGA for one compute unit for processing an image of size 512 × 512 is shown in Table 2 ▸.

Given the low resource usage, there is considerable potential to increase the throughput of the algorithm. In the current implementation, one new 8-bit pixel value is read in per clock cycle, but each internal channel of the FPGA is capable of reading 512-bit-wide vectors, which would allow pixels to be processed in 64-pixel-wide blocks. However, either additional logic would be required to handle the pixels at either end of each block or key points at the edges of blocks could be missed (which may be acceptable in practice). In terms of FPGA resources, this approach would not greatly increase the amount of RAM needed, since we would still buffer four lines of data, but processing the 64 pixels in parallel would require more resources for mathematical operations and logic. Aside from RAM, the current implementation uses around 0.5% of the FPGA’s resources, so if we make the assumption that this implementation would require 64 times more resources for a factor of 64 acceleration, this would use approximately one-third of the FPGA’s resources for an execution time of 0.03 ms.

#### 3.2.2. Generating feature vectors

As mentioned in Section 3.2, a feature vector is a numerical representation of the image that captures its relevant information for the machine learning task. In serial crystallography, the feature vector typically includes features that describe the spatial and intensity distribution of the diffraction spots and background. There are different methods for extracting features from serial crystallography images, depending on the specific application and the nature of the images. One common approach is to use image processing techniques to identify and extract the diffraction spots and their properties, such as intensity, size, shape and orientation. These features can be represented as a set of numerical values or vectors, which are combined to form the feature vector.

The robustness of the feature vector against different data sets is critical for achieving good performance in machine learning. A feature vector that is designed to be robust to a specific data set may not perform well on other data sets with different characteristics, such as the crystal orientation, the X-ray wavelength, the detector resolution and the imaging geometry. In addition, it is important to consider the interpretability and explainability of the feature vector, especially in scientific applications such as serial crystallography image classification. This can involve using feature selection techniques to identify the most informative and relevant features for the task, as well as visualizing and analysing the feature vector to gain insights into the underlying data distribution and structure.

In this paper, we divide the image into regions and count the number of key points which have been extracted by the MP-FAST algorithm in each region. To generate a feature vector based on the number of key points in each region, the key points (mostly Bragg spots) are detected in the entire image using the MP-FAST key-point detection algorithms as described above.

**Figure 15:**
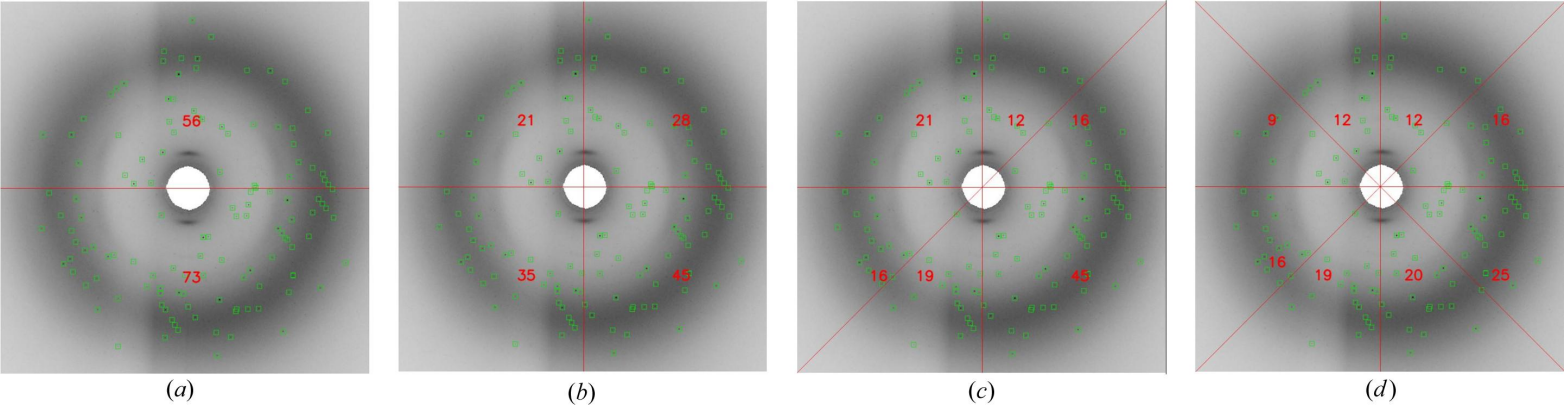
Feature vector generation by splitting the image into (*a*) two parts, (*b*) four parts, (*c*) six parts and (*d*) eight parts and counting the number of peaks in each region.

The image is then partitioned into *n* = 8 regions, and the count of key points (not peak positions) in each region is tallied. This collective count is then utilized to generate the feature vector. This ensures that the feature vector size remains consistent across all experiments. The incorporation of eight regions is driven by the necessity for a relatively symmetrical arrangement of key points. This choice aims to enhance reliability, especially in the face of challenges such as noisy patterns. Additionally, it ensures an equitable opportunity for all the diverse regions of the image to contribute to the generation of a robust feature vector. Fig. 15 ▸ visually presents various symmetrical patterns.

**Figure 16:**
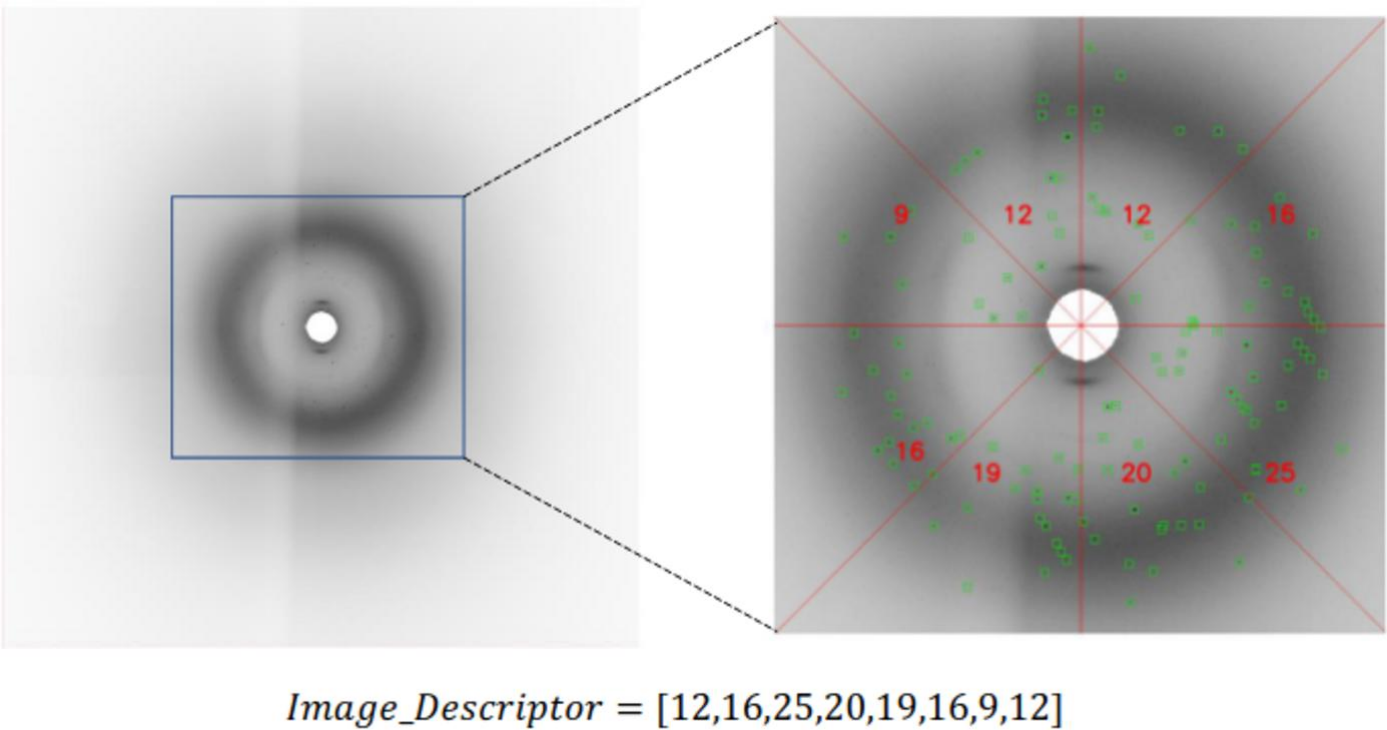
Dividing the image into eight regions and generating an image descriptor vector based on the number of detected key points.

Finally, the counts of Bragg spots in each region are combined into a single feature vector. This feature vector can then be used as input to machine learning algorithms for image classification tasks. Fig. 16 ▸ shows the image feature descriptor for eight regions.

**Figure 17:**
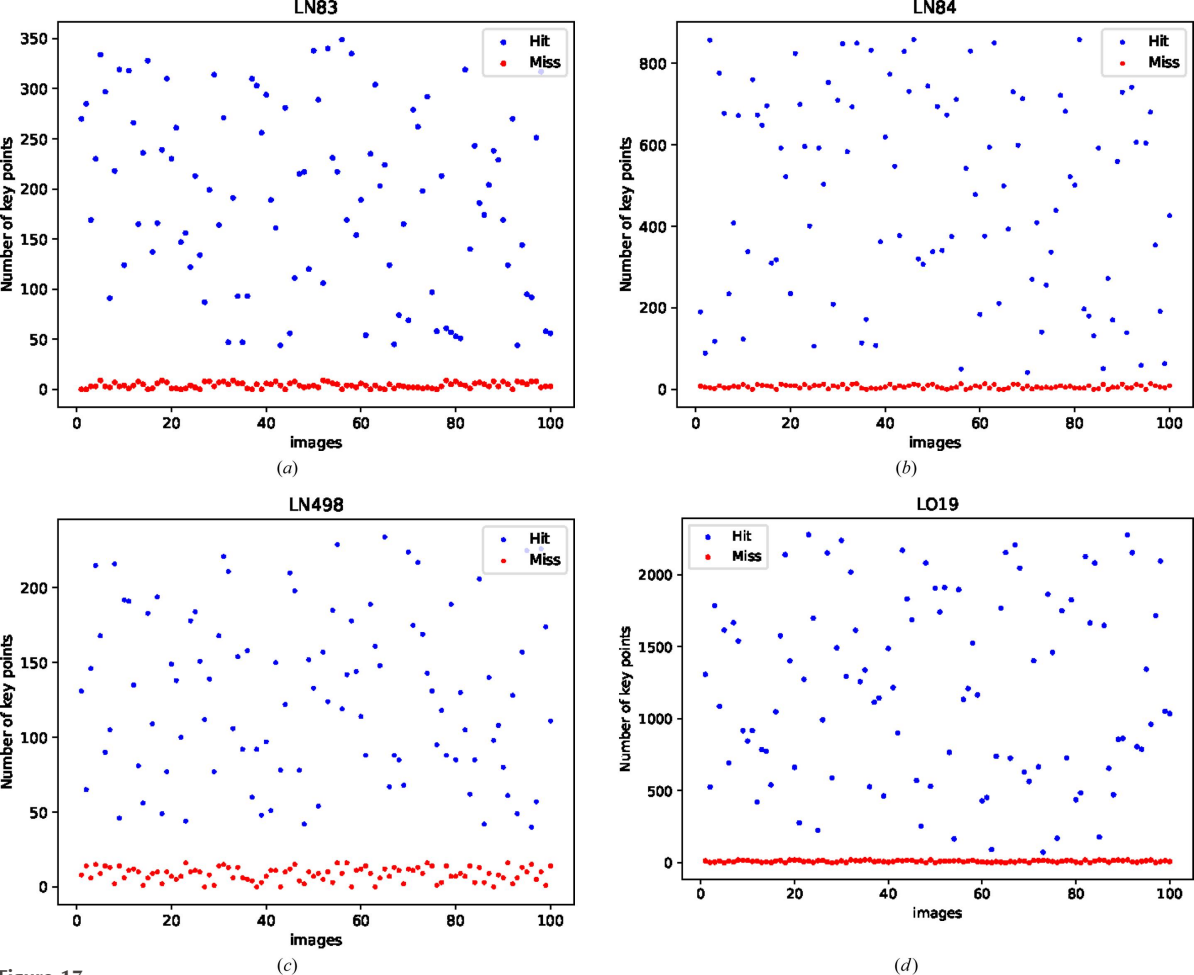
Demonstrating the number of key points detected by MP-FAST for 100 images of hit and 100 images of miss classes for four different data sets.

If a diffraction pattern does not contain any Bragg spots, this means that the beam did not intersect with a protein crystal. In the context of generating a feature vector based on key points where the Bragg spots are ROIs, the feature vector will be empty. Fig. 17 ▸ shows the number of key points detected by MP-FAST for 100 images of hit and 100 images of miss classes for four different data sets.

As is shown in Fig. 17 ▸, it is evident that for the miss images MP-FAST will not detect any key points, or may detect just a few key points, and the resulting feature vector will be sparse or empty. This can be useful for machine learning tasks that rely on the feature vector to distinguish between hit and miss diffraction patterns. By treating miss images as a separate class, the algorithm can learn to recognize patterns and features that are associated with unsuccessful diffraction patterns. This can help improve the overall accuracy and efficiency of the machine learning model.

### 3.3. Classifier

**Figure 18:**
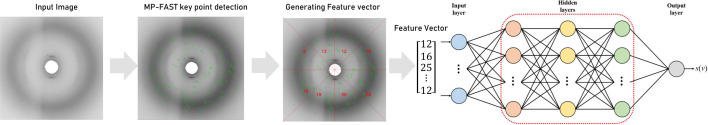
The image classification pipeline including MP-FAST key-point detection, generation of the image descriptor vector and the MLP classifier.

The classification of X-ray diffraction patterns involves two main steps. The first step involves applying the MP-FAST feature extraction technique to obtain key points, feature vectors and descriptors from labelled patterns. In the second step, a machine learning algorithm is trained to classify the images into different categories. Fig. 18 ▸ shows the image classification pipeline based on the proposed method.

In this work, we compared four supervised classifiers, namely the MLP, SVM, random forest (RF) and naïve Bayes (NB), to train the feature vectors extracted using the MP-FAST key-point detection and image descriptor technique explained in Section 3.2.2.

Our experimental results demonstrate that the MLP classifier produces superior performance across all data sets. Specifically, the MLP exhibits higher accuracy, precision and recall than the other classifiers, demonstrating its effectiveness in capturing complex relationships within the feature space.

One of the essential steps in the machine learning model is hyperparameter tuning because it directly impacts a machine learning model’s performance and generalization to new unseen data. Choosing the right hyperparameters can mean the difference between a model that underfits (too simplistic) or overfits (too complex) the data and one that achieves optimal predictive accuracy. Proper tuning ensures the model is well suited to the specific problem, leading to better results and improved real-world applicability.

We utilized the *GridSearchCV* tool from the *Sklearn* library (Pedregosa *et al.*, 2011 ▸) for hyperparameter tuning. Various hyperparameters such as hidden layer sizes, activation functions (logistic, tanh, relu), solvers (sgd, adam), alphas (0.0001, 0.05) and learning rates (constant, adaptive) were explored. Subsequently, the optimal hyperparameters were determined as follows: stochastic gradient descent (SGD) was employed for weight optimization; the MLP consisted of four hidden layers, each with *L* neurons, where *L* = {50, 30, 20, 20}; the activation function was *logistic* which returns 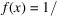 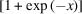 ; and the regularization term was 10^−5^.

For the RF classifier, we specified the parameters as follows: ‘criterion’ is ‘gini,’ maximum depth is 30, and the initial number of estimators is 100. For the SVM classifier, we set the regularization parameter *C* = 10 and used the *rbf* kernel. For the NB classifier we considered var_smoothing = 1 × 10^−9^, which is the portion of the largest variance of all features that is added to variances for calculation stability.

## 4. Experiments

This section covers both the experimental data and the implementation details of our pipeline. We present the experimental results and discuss the CPU, GPU and FPGA implementations for the classification task.

### 4.1. Data sets

To evaluate our pipeline, we utilized both synthetic and real experimental serial crystallography data in our experiments. The synthetic data set, named DiffraNet (Souza *et al.*, 2019 ▸), was generated using the *nanoBragg* simulator (https://bl831.als.lbl.gov/~jamesh/nanoBragg/), which employs a single-crystal structure and varying X-ray beam intensity to produce variations in image quality. Imperfections in the crystal were also modelled by breaking it up into smaller crystals. Background noise sources and crystal orientation were additional parameters considered in generating this data set. It consists of 25 000 diffraction patterns with an image size of 512 × 512 pixels, divided into five classes (Blank, No Crystal, Weak, Good, Strong) for performing the classification task.

We selected four diverse experimental data sets to reflect different imaging detectors, beam energies and sample delivery methods, as well as crystals with varying space groups and unit-cell parameters, to represent our experimental data. These protein serial crystallography diffraction data sets were collected on the Coherent X-ray Imaging (CXI) and Macromolecular Femtosecond Crystallography (MFX) instruments of the Linac Coherent Light Source (LCLS). In the recent study by Ke *et al.* (2018 ▸), the first 2000 ▸ images from the native LCLS data format were converted to a 4-byte integer HDF5 format for further analysis. We utilized the same diffraction patterns and evaluation protocols in our experiments as presented in their work. These data sets are labelled by human annotation and the diffraction integration for advanced light sources (*DIALS*) (Winter *et al.*, 2018 ▸) spotfinder. Table 3 ▸ provides a summary of the experimental data sets used. We also applied our MP-FAST pipeline to peak finding on two complete data sets recorded by an AGIPD detector, one from the well known model system in crystallography, lysozyme, and the other from a complex of a β-lactamase from *Klebsiella pneumoniae* involved in antibiotic resistance (Wiedorn *et al.*, 2018 ▸).

### 4.2. Implementation details

Our pipeline comprises feature extractor and machine learning components, whose performance we evaluate using standard classification evaluation metrics such as accuracy, precision, recall and *F*1 scores. For our experiments, we utilized the C++ programming language, along with the *OpenCV* library. For CPU and GPU implementations the experiments were run on a system with an Intel Core i7-11370 H CPU at 3.30 GHz, featuring 1 MB L2 cache, 8 MB L3 cache and 16 GB RAM. Additionally, we employed an NVIDIA RTX 3070 GPU equipped with 10 496 CUDA cores. For the FPGA implementation we ran the code on a Xilinx Alveo U280 which is equipped with 8 GB of HBM, two 16 GB DDR4 RDIMMs operating at 2400 MT s^−1^ and two QSFP28 Ethernet ports capable of 100 Gb s^−1^ each.

### 4.3. Experimental results

Here we present a comprehensive analysis of the outcomes obtained through rigorous experimentation. In subsection 4.3.1 we demonstrate the performance and accuracy of our proposed methodology in classifying diverse data sets. This involves evaluating the effectiveness of our classification models (MLP, SVM, RF and NB) against three real and one synthetic data set. We also compare the classification results for accuracy and processing time performance for various components of our proposed method with the CNN method (Ke *et al.*, 2018 ▸) and the ORB+MLP (Rahmani *et al.*, 2023 ▸) method. Subsection 4.3.2 focuses on the efficiency and acceleration achieved through parallelizing key components of our approach. We compare the performance achieved by employing the different hardware accelerators, specifically focusing on the CPU, GPU and FPGA.

#### 4.3.1. Classification performance

The MLP, SVM, RF and NB classifiers (Section 3.3) were trained using feature vectors extracted through the MP-FAST key-point detection and image descriptor technique, as elucidated in Section 3.2. Table 4 ▸ shows the classification performance of the four classifiers for the three data sets LO19, LN83 and LN84 and one synthetic data set DiffraNet for *F*1 score, precision, recall/sensitivity and accuracy with the same training and testing strategy on real experimental data.

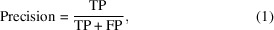




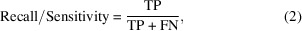




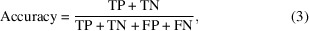




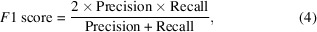

where TP denotes true positive, TN true negative, FP false positive and FN false negative.

Our experimental results demonstrate that the MLP classifier produces superior performance across all data sets. Specifically, the MLP exhibits higher accuracy, precision and recall than the other classifiers, showing its effectiveness in capturing complex relationships within the feature space.

Table 5 ▸ compares the classification results for our proposed method with the CNN (Ke *et al.*, 2018 ▸) and ORB+MLP (Rahmani *et al.*, 2023 ▸) methods for accuracy performance with the one-to-one (train and test with same data set) and cross (train by one and test with another data set) training and testing strategy on both synthetic and real experimental data, and Table 6 ▸ shows a comparison of processing time for various components of our GPU-based MP-FAST image classification method with other methods.

#### 4.3.2. Parallelization performance

Parallelization on the CPU is achieved by considering the number of kernels *n*, where *n* is the number of CPU threads. The image is divided into *n* regions and each kernel runs MP-FAST for each region. Extensive experiments showed that the execution performance time is 1.55 ms for an image of size 512 × 512 pixels, 3.03 ms for an image of size 1024 × 1024 pixels and 8.91 ms for an image of size 2048 × 2048 pixels. The acceleration achieved by parallelization increases with the number of CPU threads, demonstrating the effectiveness of our approach. Additionally, we have observed that the parallelization of the algorithm did not compromise the accuracy of feature detection.

To parallelize the MP-FAST algorithm on a GPU, we partitioned the input image into a grid of (*W* + 15)/16 × (*H* + 15)/16 blocks. Each pixel is treated by a separate thread within each block and the results are combined to obtain the final output. Extensive experiments using a high-end GPU showed that the parallelization of the MP-FAST algorithm on a GPU significantly reduces the execution time compared with the CPU-parallelized and non-parallelized versions. Specifically, the execution performance time, including data transfer from host to device and kernel execution time, is 0.360 ms for an image of size 512 × 512 pixels, 0.52 ms for an image of size 1024 × 1024 pixels and 1.4 ms for an image of size 2048 × 2048 pixels.

Our implementation of the algorithm on an FPGA, which used less than 1% of the FPGA’s resources, had an execution time of 2 ms for an image of size 720 × 720, roughly equalling the non-parallelized CPU version. We estimate that further parallelization could speed this up by a factor of approximately 64, while using roughly one-quarter of the FPGA’s resources; this would give an execution time of the order of 0.04 ms, which is slower than the GPU but would still leave resources for other processing steps.

The size of an image can have a significant impact on execution time performance for image processing, particularly for sequential CPU processing, parallel CPU processing and FPGA pipelining. Table 7 ▸ shows the runtime performance of different sizes of diffraction pattern for CPU, GPU and FPGA implementations. For sequential CPU processing, the size of the image can directly impact the execution time performance. This is because the CPU can only process one instruction at a time and must process the image pixel by pixel in a sequential manner. Therefore, the larger the image, the more time it will take for the CPU to complete the processing.

For parallel CPU processing, the size of the image can also have an impact on the execution time performance, but it may not be as significant as for sequential CPU processing. In parallel processing, multiple CPU threads can work simultaneously to process the image, which can reduce the overall execution time. However, if the image is too large to fit into the number of threads of each CPU, it may need more waiting time to synchronize the threads, which can add overhead to the processing time.

For parallel GPU processing, the size of the image can also have a significant impact on the execution time performance. GPUs are optimized for parallel processing and can process large amounts of data simultaneously. However, for parallel algorithms like the artefact detection described in Section 3.1 where 100 of the miss images are stacked, the size of the image can still impact the execution time. The image must be loaded into the GPU’s memory and, if the images are too large, they may not fit into the GPU’s memory, or there may not be sufficient free threads (one thread per pixel), resulting in an additional overhead.

For image processing on the FPGA, the size of the image can also have a significant impact on the execution time performance, but the specific impact will depend on the design of the FPGA-based image processing system.

Our experimental results show that parallelizing the MP-FAST algorithm on a GPU is a promising way of improving the performance of feature detection algorithms.

## 5. Conclusions

In this paper, we have introduced a new method called MP-FAST for detecting key points in serial crystallography images, and have described a general pipeline for data classification. We have implemented the MP-FAST algorithm on three different processors, CPU, GPU and FPGA, and analysed their performance.

Our experiments have showed that the GPU parallelization yields the best results in terms of reducing the execution time when compared with the other processors and the non-parallelized version, leading to a more efficient algorithm. Our approach has the potential to be applied to other algorithms that deal with large images. Parallelizing the MP-FAST algorithm on a GPU is a promising way of improving the performance of feature detection algorithms.

Additionally, we have evaluated our classification approach based on MP-FAST using a multi-layer perceptron on various data sets, including synthetic and experimental data, which resulted in superior performance compared with other feature extractors and classifiers.

## Figures and Tables

**Table 1:** Screening success rate of applying a CNN trained with one Rayonix data set to another Rayonix data set (Ke *et al.*, 2018 ▸) Numbers in bold indicate the best performance.

Train/test	LO19	LN83	LN84
LO19	**93%**	85%	65%
LN83	91%	**96%**	90%
LN84	74%	92%	**90%**

**Table 2:** Resource usage in the FPGA for one compute unit

Kernel(s)	LUT (% used)	Register (% used)	BRAM (% used)	URAM (% used)	DSP (% used)
mp_fast_1	7.708 (0.59%)	12.290 (0.5%)	23 (1.14%)	0 (0.0%)	37 (0.41%)

**Table 3:** Experimental data

LCLS data set (proposal, run)	Incident energy (eV)	Protein	Space group, unit cell (Å)	Instrument	Sample delivery	Detector
L498, 27	9773	Thermolysin	*P*6_1_22, *a* = 93, *c* = 130	CXI	MESH	CSPAD
LN84, 95	9516	Photosystem II	*P*2_1_2_1_2_1_, *a* = 118, *b* = 223, *c* = 311	MFX	Conveyor belt	Rayonix
LN83, 18	9498	Hydrogenase	*P*2_1_2_1_2_1_, *a* = 73, *b* = 96, *c* = 119	MFX	Conveyor belt	Rayonix
LO19, 20	9442	Cyclophilin A	*P*2_1_2_1_2_1_, *a* = 42, *b* = 52, *c* = 88	MFX	Liquid jet	Rayonix

**Table 4:** Classification performance (%) of four classifiers trained on MP-FAST features for real and synthetic data sets

Classifier	*F*1	Precision	Recall	Accuracy
LO19				
MLP	94.88	95.26	95.17	95.04
SVM	92.89	93.52	91.43	92.13
RF	91.62	89.62	90.33	90.43
NB	91.40	91.33	91.42	91.54
				
LN83				
MLP	94.15	93.84	93.94	94.07
SVM	92.63	91.42	93.01	92.53
RF	93.42	93.49	93.46	93.15
NB	92.29	93.61	92.00	92.88
				
LN84				
MLP	96.42	96.33	97.63	97.38
SVM	91.72	91.39	93.27	92.77
RF	88.72	87.42	88.41	88.38
NB	89.21	89.91	89.42	89.67
				
DiffraNet				
MLP	97.19	97.55	97.11	97.21
SVM	96.77	97.03	96.64	96.37
RF	94.62	93.92	94.83	94.77
NB	94.54	94.57	94.73	94.02

**Table 5:** Classification performance (%) with cross-data-set training and testing for our proposed method compared with the CNN method (Ke *et al.*, 2018 ▸) and ORB+MLP (Rahmani *et al.*, 2023 ▸)

Train/test		LO19	L498	LN83	LN84
LO19	MP-FAST	95.04	87.74	91.22	93.26
	CNN	93		85	65
	ORB	92.0	73.3	75.7	77.5

L498	MP-FAST	87.12	93.77	87.58	86.44
	CNN		82		
	ORB	69.7	89.7	71.5	67.1

LN83	MP-FAST	87.8	87.91	94.07	91.88
	CNN	91		96	90
	ORB	72.3	78.4	93.5	80.1

LN84	MP-FAST	92.21	86.81	93.31	97.38
	CNN	74		92	90
	ORB	79.1	75.9	72.6	96.5

**Table 6:** Processing time (ms) for various components of our proposed method compared with the CNN method (Ke *et al.*, 2018 ▸) and the ORB method (Rahmani *et al.*, 2023 ▸) on 64 images

	CNN			ORB				MP-FAST		
	LCN	CNN	Total time	ORB	BVWs	MLP	Total time	MP-FAST	MLP	Total time
Train	5700	260	5960	11.264	3171	197.3	3379.564	24.31	188.5	212.81
Test	5700	50	5750	11.584	3099	101.03	3211.614	23.88	101.7	125.58

**Table 7:** Execution time performance for feature extraction (in milliseconds)

	Image size			
Hardware	512 × 512	720 × 720	1024 × 1024	2048 × 2048
CPU, sequential	2.13	4.88	8.71	15.23
CPU, parallel	1.55	2.38	3.17	8.91
FPGA	1.00	2.01	3.02	14.00
GPU	0.36	0.41	0.49	0.83
